# Gastroprotective Activity of the Total Flavones from *Abelmoschus manihot* (L.) Medic Flowers

**DOI:** 10.1155/2020/6584945

**Published:** 2020-02-24

**Authors:** Jun Zhang, Zai-Lin Fu, Zhao-Xing Chu, Bi-Wei Song

**Affiliations:** ^1^Department of Pharmacy, The Second Affiliated Hospital of Zhejiang Chinese Medicine University, Hangzhou, Zhejiang 310005, China; ^2^Institute of Pharmacology, Zhejiang University of Technology, Hangzhou, Zhejiang 310014, China; ^3^Department of Pharmacy, First People's Hospital of Yuhang District, Hangzhou, Zhejiang 311100, China; ^4^China Pharmaceutical University, Nanjing, Jiangsu 210009, China

## Abstract

**Background:**

*Abelmoschus manihot* (L.) Medic flower is a medicinal plant for the treatment of diseases in China. The present study was carried out to scientifically validate the gastroprotective activity and clarify the possible mechanism of the total flavones from *Abelmoschus manihot* (L.) Medic flower is a medicinal plant for the treatment of diseases in China. The present study was carried out to scientifically validate the gastroprotective activity and clarify the possible mechanism of the total flavones from

**Methods:**

Gastric ulcer was induced in mice by oral administration of ethanol. The gastroprotective activity of TFA was evaluated by the gastric ulcer index and histological examinations. The gastric tissue was collected in the form of homogenate. The level of malondialdehyde (MDA) and glutathione (GSH), the activity of superoxide dismutase (SOD), and protein content were measured. Western blotting for the expression of Bax, Bcl-2, TNF-*α*, and NF-*κ*B(p65) was also carried out. The effect of TFA was compared with that of standard antiulcer drug omeprazole (100 mg/kg).

**Results:**

This gastroprotective effect of TFA could be attributed to the increase in the activity of SOD and GSH and decrease in the levels of MDA and also decrease in the levels of Bax, TNF-*α*, and NF-*κ*B(p65) was also carried out. The effect of TFA was compared with that of standard antiulcer drug omeprazole (100 mg/kg).

**Conclusion:**

The findings of this study demonstrated that TFA could significantly attenuate ethanol-induced gastric injury via antioxidative, anti-inflammatory, and antiapoptotic effects.

## 1. Background

Gastric ulcer is a common digestive disease with an increasing incidence and prevalence attributed to the loss of balance between aggressive and protective factors [1]. There is about 10% of the world's population suffering from the gastric ulcer [2]. The most common presenting symptom of patients with the gastric ulcer is often characterized with burning or gnawing pain in the center of the abdomen [3].

The etiology of the gastric ulcer is multifactorial, including alcohol abuse, stress, smoking, nonsteroidal anti-inflammatory drugs overuse, and *Helicobacter pylori* infection [4, 5]. Excessive alcohol consumption is a major reported causesr cause for gastric ulcer [6]. Alcohol, as an ulcerogenic substance, can perturb the gastric mucosa and induce metabolic disorders, leading to inflammation, hyperemia, edema, hemorrhage, and erosive lesions [7]. Alcohol also markedly increases the accumulation of free radical oxygen and lipid peroxidation and synchronously suppresses the activity of antioxidant enzymes such as superoxide dismutase and glutathione peroxidase in the gastric tissues, which promotes the necrosis and apoptosis of gastric epithelial cells [8]. Thus, alcohol is commonly used to induce experimental gastric ulcer models in animals [9]. Most of the existing antiulcer drugs mainly work via the manner of acid suppression and indirectly promote the self-healing of gastric mucosa, but show limited efficacy [10]. Lots of clinical and experimental studies have demonstrated that utilization of herbal medicines could be a valuable alternative to cure the gastric ulcer with few adverse effects [11].

*Abelmoschus manihot* (L.) Medic is a medicinal plant which belongs to the family of Malvaceae. It is widely distributed in Papua New Guinea, New Caledonia, and China [12]. As a folk medicine in Eastern Europe and Asia, its flower has been used for a variety of purposes for hundreds of years [13]. Pharmacological researches have demonstrated that the extractive of its flowers has a wide range of pharmacological activities, including analgesic, anti-inflammory, antioxidant, hepatoprotective, renal protection, and cardiovascular protection [14]. The extensive phytochemical and pharmacological studies have indicated that total flavonoids extracted from flowers of *Abelmoschus manihot* (L.) Medic (TFA) were the major bioactive compounds in the extractive [15, 16]. However, no systematic study has been carried out on TFA to verify for the gastroprotective activity yet. Thus, this work was undertaken to investigate and validate the protection effect of TFA on gastric ulcer in mice and its possible mechanism of action.

## 2. Materials and Methods

### 2.1. Drugs and Reagents

Omeprazole was purchased from AstraZeneca Pharmaceutical Co., Ltd. (London, UK). GSH, MDA, and SOD kits were provided by Jiancheng Bioengineering Institute (Nanjing, China). GAPDH antibody, Bcl-2, Bax, NF-*κ*B p65, and HRP-conjugated secondary antibody were purchased from Boster (Wuhan, China). TNF-*α* antibody was provided by Proteintech Group Inc. (Chicago, USA). Hyperoside standard was provided by Anhui Institute of Medical Science. All other chemicals were of analytical-reagent grade with the highest quality commercially available.

### 2.2. Plant Material and TFA Preparation

The dried flowers of *Abelmoschus manihot* (L.) Medic were purchased from the wholesale market of Chinese Herbals in Bozhou, Anhui Province. The flowers were identified by Prof. Song Biwei (College of Pharmaceutical Sciences, Zhejiang University of Technology). A voucher specimen was deposited in the Herbarium of the Department of Pharmacology of the College of Pharmaceutical Sciences, Zhejiang University of Technology.

The TFA was extracted from the flowers of *Abelmoschus Manihot* (L.) Medic by the Department of Pharamcology, Zhejiang University of Technology, Zhejiang, China. The dried flowers were shredded in an electric mill. Powdered *Abelmoschus Manihot* (L.) Medic flowers were immersed in 70% ethanol (v/v). The mixture was extracted 3 times under reflux for 0.5 h. Then, the decoction was filtered through the analytical filter paper and evaporated by rotary evaporation under vacuum at 40°C. The “drug extract” ratio of *Abelmoschus manihot* ethanolic extract was 1: 1 (1 g/mL). The main active components of TFA were hyperoside, isoquercitrin, hibifolin, quercetin-30-O-glycoside, quercetin, myricetin, and rutin [13].

### 2.3. Total Flavonoids Content Determination

Total flavonoids content of the decoction was determined by the Acetic Acid-Sodium Acetate-Aluminum Chloride method with some modifications [17]. Briefly, the decoction was diluted to 25 mL using 70% ethanol. 1 ml of the diluted ethanolic extract was transferred to a 25 mL volumetric flask. 5 mL of Acetic Acid-Sodium Acetate buffer (2 M Acetic Acid: 2 M Sodium Acetate buffer = 3 : 1) was added to the mixture followed by adding 3 mL of 0.1 M Aluminum Chloride solution. The final volume was adjusted to 25 ml by ultrapure water. The mixture was incubated at room temperature for 40 min, and the absorbances of the mixture were measured in 401 nm. The standard hyperoside curve was used to calculate total flavonoid content. The analysis was performed in triplicate.

### 2.4. Hyperoside Content Determination by HPLC

Samples for HPLC were prepared from the decoction. 0.5 mL of the TFA was transferred to a 25 mL volumetric flask. The solutions were diluted to 25 mL by HPLC grade methanol. They were filtered through a 0.2 *μ*m syringe filter. Reference standard hyperoside was prepared by weighing 0.1 mg/ml of hyperoside. A Hitach L2000 series HPLC system (Hitach, Tokyo, Japan) was used to analyze hyperoside in this experiment. The analytical column used was a 250 mm × 4.6 mm i.d. HITACHI LaChrom C18 (5 *μ*m) maintained at 30°C. The flow rate was kept at 1 ml/min, and the injection volume was 10 *μ*L. Two solvents, A (acetonitrile) and B (0.1% phosphoric acid) were used for elution of constituents. The elution of the mobile phase can be described as follows: (A)/(B) = 10/90 (0 min) ⟶ 20/80 (20∼30 min) ⟶ 25/75 (30∼40 min) ⟶ 30/70 (40∼45 min) ⟶ 15/85 (45∼50 min) ⟶ 10/90 (50∼60 min). The chromatogram peaks were detected at 360 nm [18].

### 2.5. Experimental Animals

The experiments were carried out on healthy ICR mice (18–22 g, 6–8 weeks) of either sex. They were obtained from Zhejiang Academy of Medical Sciences (License no. SCXK 2014-0001). All mice were kept in standardized animal house at 25 ± 2°C, light and dark cycles of 12/12 hours, respectively. A balanced diet and free access of water were provided. They were starved for 24 hours before use though water was allowed *ad libitum*. The mice were cared for in accordance with the “Principles of laboratory animal care” (NIH publication no. 82–23, revised 1996) guidelines. The experiments were designed according to the Institutional Animal Ethical Committee.

### 2.6. Ethanol-Induced Gastric Ulcer Model

The experiment was carried out according to the method of Hollander et al. with a few modifications [19]. The mice were randomly divided into 6 groups of 10 mice each: control group, model group, omeprazole group (100 mg/kg), and TFA group (300, 600, and 1200 mg/kg). They were all gavaged with 0.4 mL absolute alcohol except the control group. The same volumes of sterile water were gavaged to the mice of the control group. 1 hour later, sterile water, omeprazole, or TFA was administrated to each group. After 4 hours, they were sacrificed by cervical dislocation.

### 2.7. Determination of Ulcer Index and Percentage of Inhibition

The isolated stomachs were immediately fixed with 4% paraformaldehyde for 20 minutes. Then they were opened along the greater curvature and washed with saline solution (0.9% NaCl). The inner surface of the stomachs was examined for ulceration with the help of a dissecting microscope. The ulcer index (UI) was calculated as described by the method of Guth [20]. The percentage of inhibition was calculated by the following formula [21]:

(1) $$\begin{array}{c} \left[\frac{\left(\text{UIcontrol}-\text{UItreated}\right)}{\text{UIcontrol}}\right]\times 100. \end{array}$$

### 2.8. Biochemical Estimation

The tissues from stomachs were cut into pieces. The weight of each one was then recorded. The samples were homogenized in 9 volumes of cold saline solution. The homogenates were centrifuged at 10000 g for 20 min at 4°C. The supernatants were used to determine the biochemical parameters. The concentration of total protein in the supernatants was measured by the BCA protein assay kit (Boster, Wuhan, Hubei, China). Levels of GSH, MDA, and SOD were determined by the commercial assay kits according to the manufacturer's instructions (Jiancheng Bioengineering Institute, Nanjing, China) for users.

### 2.9. Histopathological Evaluation

One part of the stomachs was fixed in 4% paraformaldehyde solution for 24 hours. Then, they were embedded in paraffin wax. The 5 *μ*m thickness tissue sections were repaired and stained with hematoxylin and eosin (HE). The slides were examined under light microscope to observe the morphological changes and recorded.

### 2.10. Western Blotting

Gastric tissues were cut into pieces and homogenized with ice-cold cell lysis buffer for western blotting and an IP kit (Beyotime, shanghai, China) for 30 min at 4°C. The homogenates were centrifuged at 14000 g for 10 min at 4°C. Then, the supernatants were collected and the concentrations of total protein were detected by a BCA protein assay kit (Boster, Wuhan, Hubei, China). Equivalent amounts of proteins were separated by electrophoresis on 12% and 5% sodium dodecylsulfate (SDS) polyacrylamide gels (SDS-PAGE) and transferred to nitrocellulose membranes (Beyotime, shanghai, China). The membranes were blocked with 5% BSA at room temperature for 2 hours. The blots were incubated overnight at 4°C with primary antibodies against TNF-*α* (1 : 1000, Proteintech Group Inc, Chicago, USA), Bax, Bcl-2, GAPDH, and NF-*κ*B p65 (1 : 200, Boster, Wuhan, China). After washing, the membranes were incubated with the appropriate HRP-conjugated secondary antibodies (Boster, Wuhan, Hubei, China) for 2 hours at room temperature. Photodensity analysis was utilized with the chemiluminescence detection system (Bio-Rad, Hercules, CA, USA) after visualizing by HRP substrate ECL solution (Boster, Wuhan, China).

### 2.11. Statistic Analysis

The results of the experiments were presented as mean ± SD, and one-way analysis of variance (ANOVA) was used to analyze comparison in different groups. *p* < 0.05 was considered to be significant. Data analysis was achieved using the software progam GraphPad Prism 5.

## 3. Results

### 3.1. Total Flavonoids Content

After making a standard calibration curve by hyperoside (*y* = 0.0246*x* − 0.0023, *r*^2^ = 0.9999), the total flavonoids content of the TFA was found to be (4.68 ± 0.07)% (w/w), respectively.

### 3.2. Hyperoside Content

**Figure 1:**
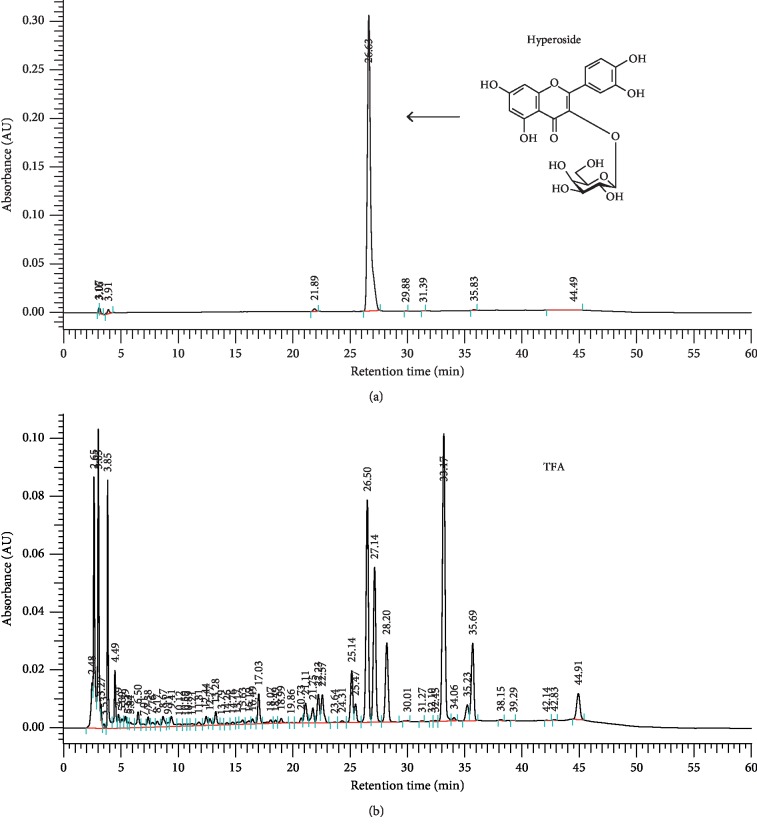
HPLC profile of hyperoside and TFA.

The standard calibration curve of hyperoside was used to evaluate the content of flavonoid in the extract. The hyperoside content of the TFA was (1.14 ± 0.27)% (w/w), respectively (Figure 1).

### 3.3. Effect of TFA on the Ulcer Index and Percentage of Inhibition

**Figure 2:**
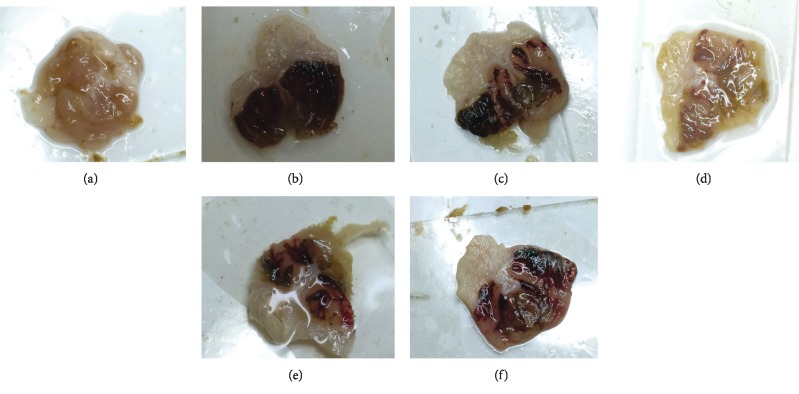
The gastric mucosal injury in mice (*n* = 10). (a) Control group; (b) model group; (c) omeprazole group; (d) high-dose group; (e) middle-dose group; (f) low-dose group.

Ethanol is regarded as one of the major risk factors for the pathogenesis of gastric ulcer. A representative stomach of each group is shown in Figure 2. The model group presented severe mucosal injury (ulcer index: 41.10 + 18.28). TFA could significantly increase the inhibition rate of ethanol-induced gastric ulcer at the dose of 600 and 1200 mg/kg (Table 1). Compared with the positive control group, TFA showed better results than omeprazole. The effect of TFA was in a dose-dependent manner, of which the high-dose group reached the maximum 66.91% inhibition rate (*p* < 0.001).

### 3.4. Effect of TFA on Biochemical Parameters

**Figure 3:**
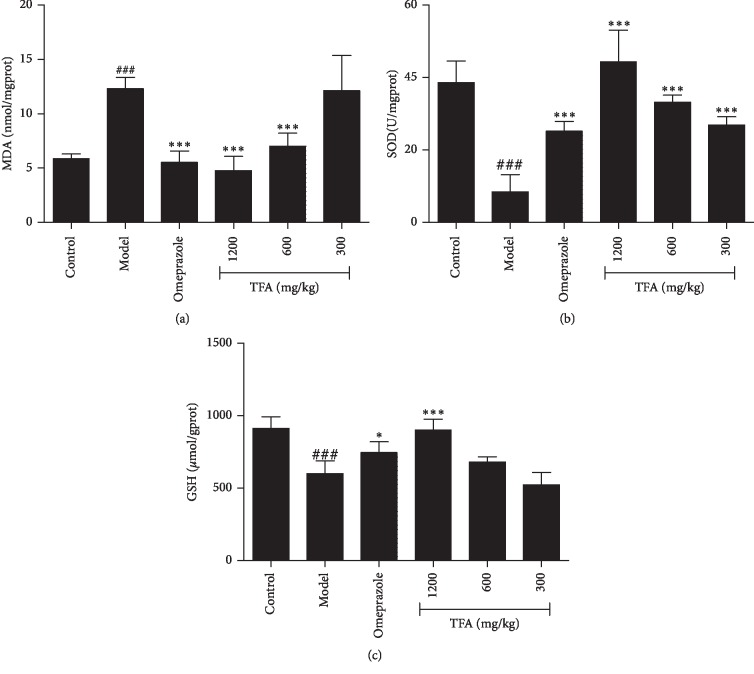
Effect of TFA on biochemical parameters of gastric tissue in mice with GU. (a) MDA levels of mice gastric tissue; (b) SOD activities of mice gastric tissue; and (c) GSH levels of mice gastric tissue (Mean ± SD, *n* = 6) ^###^*p* < 0.001 vs. control; ^*∗*^*p* < 0.05, ^*∗∗*^*p* < 0.01, ^*∗∗∗*^*p* < 0.001 vs. model.

All the results are shown in Figures 3(a)–3(c)). Ethanol caused a marked significant increase in the levels of MDA (*p* < 0.001) and reduction in the activities of SOD (*p* < 0.001) and GSH levels (*p* < 0.001) in ulcerated mice as compared to the control group. Contrarily, TFA (600 mg/kg) and TFA (1200 mg/kg) treatments dramatically decreased MDA levels in gastric tissue with a concomitant increase in the activities of SOD and GSH levels as compared with the model group, especially the high-dose group.

### 3.5. Histologic Evaluations of Gastric Tissue

**Figure 4:**
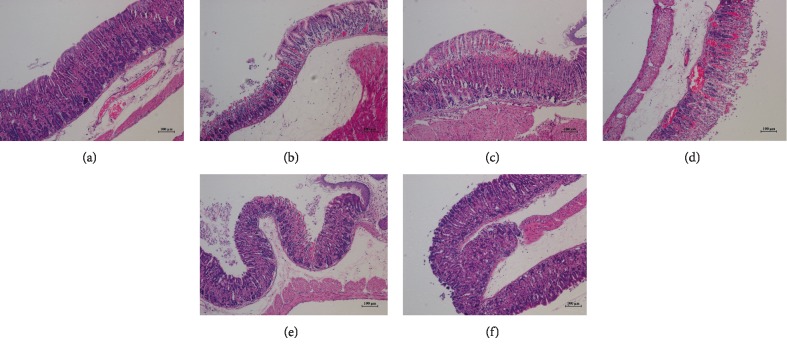
Histologic evaluations of gastric tissue (HE staining, ×100). (a) Control group; (b) model group; (c) omeprazole group; (d) low-dose group; (e) middle-dose group; and (f) high-dose group.

**Figure 5:**
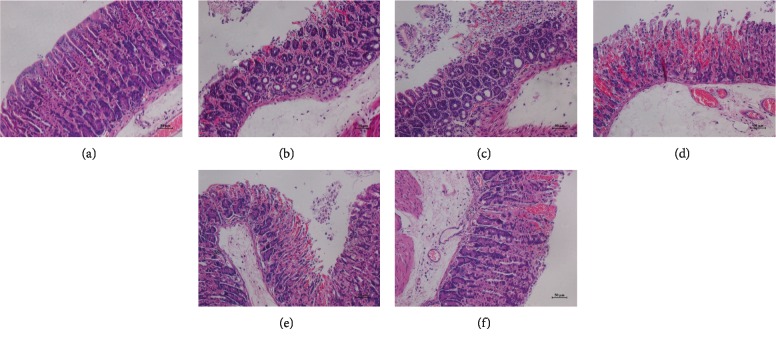
Histologic evaluations of gastric tissue (HE staining, ×200). (a) Control group; (b) model group; (c) omeprazole group; (d) low-dose group; (e) middle-dose group; and (f) high-dose group.

The gastric tissue specimens of different groups were observed under a microscope, and the pathological changes are shown in Figures 4 and 5. In the control group, the structure of the gastric tissue was intact and the epithelial cells were tightly arranged (Figures 4(a), 5(a)). However, there were various histopathological alterations including congestion, cell-contained hyperaemia and edema, gastric epithelial cells necrosis, inflammatory changes, and erosions in the gastric tissue of model group mice (Figures 4(b), 5(b)). Compared with the model group, this poor sanitation was restrained by TFA or Omeprazole, and the structure was tended to be normal. The results suggested that TFA could reduce gastric mucosa injury induced by ethanol.

### 3.6. The Expression of Proteins in the Gastric Tissue

#### 3.6.1. Effect of TFA on the Expression of Bax and Bcl-2

**Figure 6:**
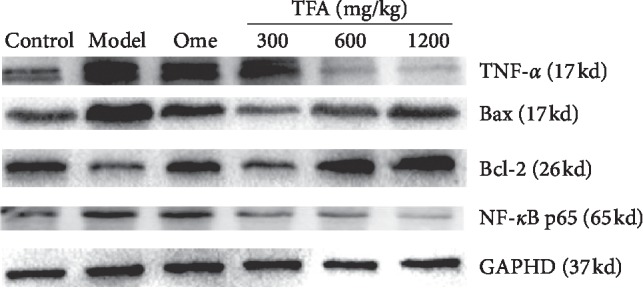
Expression of the proteins with western blot.

**Figure 7:**
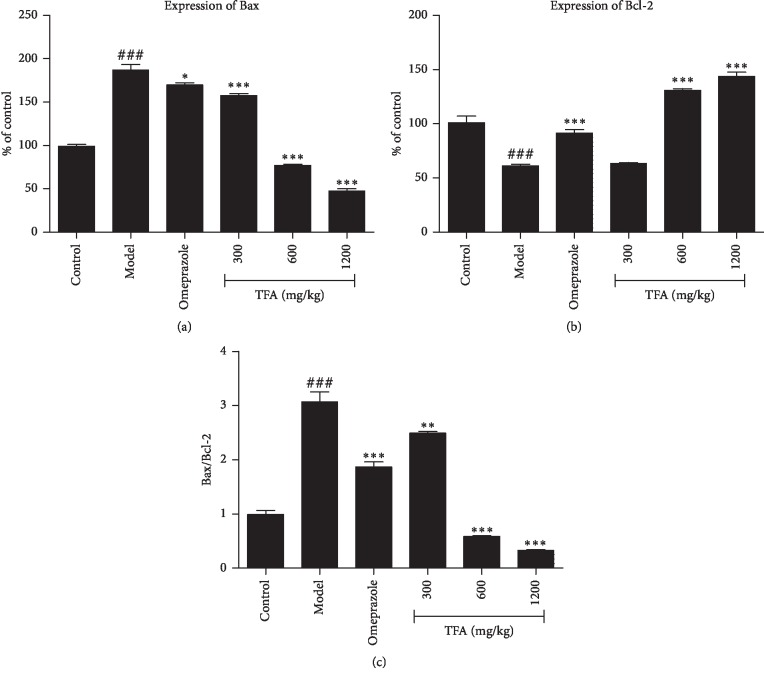
Analysis of Bax and Bcl-2 protein expression with western blot (Mean ± SD). (a) Expression of Bax; (b) expression of Bcl-2; and (c) Bax/Bcl-2 ^###^*p* < 0.001 vs. control group; ^*∗*^*p* < 0.05, ^*∗∗*^*p* < 0.01, ^*∗∗∗*^*p* < 0.001 vs. model group.

The results of Bax and Bcl-2 protein expression were shown in Figures 6 and 7. The expression of the proapoptotic factor Bax in the model group was significantly higher than the control group (*p* < 0.001), 1.87 folds higher than the control group, and the expression of the antiapoptotic factor Bcl-2 was significantly lower (*p* < 0.001), 62% of the control group. The ratio of Bax/Bcl-2 in the each treatment group was significantly lower than that in the model group, especially in the TFA high-dose group. This results demonstrated that TFA can downregulate the expression of Bax, upregulate the expression of Bcl-2, and thus decrease the ratio of Bax/Bcl-2 to inhibit the activation of the endogenous apoptotic pathway.

#### 3.6.2. Effect of TFA on the Expression of TNF-*α*, NF-*κ*B p65

**Figure 8:**
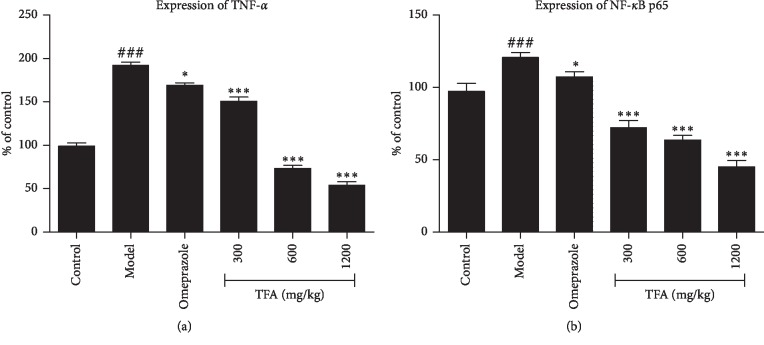
Analysis of TNF-*α* and NF-*κ*B p65 protein expression with Western blot (Mean ± SD). (a) expression of TNF-*α* (b) expression of NF-*κ*B p65 ^###^*p* < 0.001 vs. control group; ^*∗*^*p* < 0.05, ^*∗∗*^*p* < 0.01, ^*∗∗∗*^*p* < 0.001 vs. model group.

The results of the expression of TNF-*α* and NF-*κ*B p65 in gastric tissue are shown in Figures 6 and 8. The expression of TNF-*α* and NF-*κ*B p65 in the TFA group was significantly lower than that in the model group, especially in the high-dose group (*p* < 0.001). The results suggested that TFA could significantly downregulate the expression of inflammation-related proteins in the gastric tissue of gastric ulcer mice.

## 4. Discussion

In the present study, the model of ethanol-induced gastric ulcer was used to evaluate the gastroprotective activity of TFA. Ethanol is widely used in experimental gastric ulcer models because of its disadvantages. The model is simple and repeatable. The morphology, histological features, healing, and recurrence processes of ulcer are similar to human [22]. The severity of gastric injury was determined by ulcer index, and we found that TFA could inhibit the occurrence of ulcers and reduce the area of ulceration. It was shown by HE staining that TFA could relieve the pathological changes of gastric ulcer tissue in mice.

Oxidative stress is an important etiological factor in the damaged gastric mucosa [3]. GSH is a major antioxidant by interaction with lipid hydroperoxides. The ulcerogenic activity is related to the tissue levels of GSH. Increase in the levels of GSH can inhibit gastric ulceration [23]. MDA is the end-product of lipid peroxidation, and it is also an important marker of oxidative damage. The increase in MDA content is associated with the necrosis and apoptosis of cells [24]. SOD can exert its effects by removing oxygen free radicals from the body [25]. Therefore, GSH, MDA, and SOD were measured in this study to evaluate oxidative stress levels. Through the detection of the abovementioned oxidative damage indicators, we found TFA can increase the activity of SOD, the concentration of GSH, and at the same time decrease the level of MDA in gastric tissue.

Bcl-2 family proteins is a family of evolutionarily related proteins, which derives its name from B-cell lymphoma 2 [26]. There are a total of 25 genes in the Bcl-2 family known to date. Bcl-2 family proteins are pivotal regulators of apoptosis by controlling mitochondrial outer membrane permeabilization (MOMP) [27]. When the cell is stimulated by apoptotic signals, the structure of Bax changes, shifting from the cytoplasm to the mitochondrial membrane and recruiting an amount of Bax proteins to form a polymer. Then, the polymer is inserted into the outer mitochondrial membrane, causing formation of mitochondrial apoptosis-induced channel (MAC). CytC, AIF, and other proapoptotic proteins release into the cytoplasm via MAC, resulting in apoptotic cell death [27, 28]. A heterodimer may be formed by the proapoptotic protein and the antiapoptotic protein, and the ratio of Bax/Bcl-2 in the dimer is directly related to the regulation of apoptosis [29]. TFA can significantly upregulate the expression of antiapoptosis protien Bcl-2 and downregulate the expression of proapoptosis factor Bax. Thus, the apoptosis of cell was prevented through downregulation of Bax/Bcl-2 ratio.

Increasing evidence suggests that inflammatory mediators may play a role in the development and progression of gastric ulcer. A lot of literature had reported that TNF-*α*, the main proinflammatory cytokine, was the important contributing factor in intestinal mucosal injury [9, 30]. TNF-*α* is the initiator of the exogenous death pathway. It can combine with autologous, proximate, or distant cell membrane death receptor (TNFR). The initiation of the death receptor pathway leads to apoptosis (activated caspase-8), programmed necrosis (via RIP1 and RIP3), or inflammation (via NF-*κ*B) responses. NF-*κ*B, a classic proinflammatory transcription factor, is kept inactive in resting cells by binding with a member of the IKB *α* inhibitor protein family [31]. NF-*κ*B is an ideal target for the mediation of proinflammatory factor expression in gastric ulcer [32]. Many natural products that have been promoted to have anti-inflammatory activity have also been shown to inhibit NF-*κ*B. In the current study, western blot analysis indicated the significant downregulation of TNF-*α* and NF-*κ*B p65 expressions in the gastric tissue, which evidenced the anti-inflammatory effect of TFA.

In summary, the present study suggested that TFA could significantly attenuate ethanol-induced gastric injury via antioxidative, anti-inflammatory, and antiapoptotic effects. Our data reinforced the therapeutic potential of TFA in the management of gastric ulcer.

## Figures and Tables

**Table 1:** Ulcer index and inhibition rate of ulceration (Mean ± SD, *n* = 10).

Groups	Dose (mg/kg)	Ulcer index	Inhibition ration (%)
Control	—	—	—
Model	—	41.10 + 18.28	—
Omeprazole	100	28.30 + 5.83	31.14
High-dose	1200	13.40 + 8.60^*∗∗∗*^	66.91
Midium-dose	600	24.10 + 12.10^*∗*^	42.09
Low-dose	300	40.30 + 17.84	1.95

*Note.* ^*∗*^ *p* < 0.05, ^*∗∗*^*p* < 0.01, ^*∗∗∗*^*p* < 0.001 vs. model group.

## Data Availability

The data used to support the findings of this study are included within the article.
